# Validation of tumour-negative lymph node size as a novel independent prognostic biomarker in oesophagogastric adenocarcinoma: *post hoc* analysis of the UK MRC OE05 and ST03 randomised trials

**DOI:** 10.1016/j.esmoop.2026.108348

**Published:** 2026-08-12

**Authors:** E. Budginaite, M. Kloft, D.R. Magee, S. Jolani, T. Goetze, M.G. Nankivell, R.E. Langley, D. Cunningham, W.H. Allum, H.C. Woodruff, H.I. Grabsch

**Affiliations:** 1 Department of Pathology, GROW – Research Institute for Oncology and Reproduction, Maastricht University Medical Center+, Maastricht, The Netherlands; 2 Department of Precision Medicine, GROW – Research Institute for Oncology and Reproduction, Maastricht University, Maastricht, The Netherlands; 3 Krankenhaus Nordwest, University Cancer Center (UCT) Frankfurt, Frankfurt, Germany; 4 The Frankfurt Institute of Clinical Cancer Research (IKF), Frankfurt, Germany; 5 School of Computer Science, University of Leeds, Leeds, UK; 6 Department of Methodology and Statistics, School of Public Health and Primary Care, Maastricht University, Maastricht, The Netherlands; 7 UCL Innovative Clinical Trials Unit, UCL, London, UK; 8 Gastrointestinal and Lymphoma Unit, Royal Marsden Hospital, London, UK; 9 Department of Surgery, Royal Marsden Hospital, London, UK; 10 Pathology and Data Analytics, Leeds Institute of Medical Research at St James’s, University of Leeds, Leeds, UK

**Keywords:** nonmetastatic lymph node size, oesophagogastric cancer, pigment, survival

## Abstract

**Background:**

The size of tumour-negative regional lymph nodes (LNnegs) may be associated with the host antitumour immune response in patients with oesophagogastric adenocarcinoma (OGAC). In the OE02 trial, larger LNnegs were independently associated with improved overall survival (OS); however, the prognostic relevance of LNneg size has not been validated in an independent OGAC population.

**Patients and methods:**

Resection specimens from 1367 patients from two phase III trials (OE05, n = 652; ST03, n = 715) were analysed. The largest LNneg per patient was identified on digitised slides stained with haematoxylin–eosin using an automated segmentation pipeline. Lymph node ratio [LNratio; number of metastatic lymph nodes (LNs) divided by the total number of resected LNs] was calculated for patients with stage ypN1+ cancer. Anthracotic pigment burden was quantified using a deep learning model. Associations among LNneg size, primary tumour location, recurrence, and survival were evaluated.

**Results:**

LNneg size and pigment burden differed by primary tumour location. LNnegs from oesophageal/junctional cancers were larger and had greater pigment burden than those from gastric cancers (all *P* < 0.0001). Larger LNnegs were associated with a lower recurrence rate (*P* = 0.033) and lower LNratio (*P* < 0.0001). Patients with LNnegs with a long axis diameter >10 mm had improved OS [hazard ratio (HR) 0.81, 95% confidence interval (CI) 0.70-0.93, *P* = 0.0147) and progression-free survival (HR 0.80, 95% CI 0.70-0.92, *P* = 0.0092). Presence of large LNnegs was independently prognostic after multivariable adjustment.

**Conclusions:**

This study validates LNneg size as a novel independent prognostic biomarker in patients with locally advanced OGAC treated with cytotoxic chemotherapy and surgery. Variation in LNneg size and pigment burden by primary tumour location may suggest anatomical or microenvironmental differences in LNneg biology that may affect LN assessment and immune-related characteristics. Further studies integrating quantitative microarchitectural and immune profiling of LNnegs are warranted to clarify the mechanisms driving LNneg enlargement and the potential functional relevance of pigment deposition.

## Highlights

• Tumour-negative lymph nodes from oesophageal cancers are larger and more pigment-laden than those from gastric cancers.
• LNneg size >10 mm is associated with improved overall survival.
• Larger LNnegs are associated with a lower LNpositive/LNtotal ratio.
• This work validates LNneg size as a novel independent prognostic factor in oesophagogastric cancer.

## Introduction

Despite advances in perioperative cytotoxic chemotherapy and, more recently, immunotherapy, long-term survival of patients with locally advanced resectable oesophagogastric adenocarcinoma (OGAC) remains limited.^1^

We and others have shown that lymph node (LN) status is the strongest prognostic factor in patients with oesophagogastric cancer.^2^ Because of the strong link to survival, the number of tumour-positive LNs is part of the tumour–node–metastasis (TNM, 7th ed)^3^ classification and used for treatment decisions. However, tumour-negative regional LNs (LNnegs) have received far less attention as potential clinically useful biomarker.

The morphological characteristics of LNnegs may reflect systemic host antitumour immune competence and represent a biomarker with potential relevance for individual patient prognostication and treatment stratification. Our group previously reported that in the UK Medical Research Council (MRC) OE02 trial, patients with locally advanced resectable oesophageal cancer and larger LNnegs in the resection specimen had better overall survival (OS), independent of established clinicopathological prognostic factors and irrespective of being treated with surgery alone or neoadjuvant cytotoxic chemotherapy followed by surgery.^4^^,^^5^ The OE02 trial included patients with squamous cell carcinoma or adenocarcinoma located in the upper, middle, or lower oesophagus. In this context, it is important to realise that the lymphatic drainage of the oesophagus, oesophagogastric junction (OGJ), and stomach differs considerably, which may influence the biological characteristics of the regional LNs. The lymphatic drainage of the stomach is multidirectional, whereas that of the oesophagus is predominantly longitudinally orientated and has direct continuity with the tracheobronchial system.^6^ In addition to draining tumours, regional LNs may also accumulate nontumoural particles such as anthracotic pigment,^7^ particularly within mediastinal LNs that collect lymph fluid originating from the lungs.^8^

It is unclear whether the observed association of LNneg size with prognosis is maintained in patients treated with different preoperative or perioperative chemotherapy regimens and in cancers located at the OGJ or within the stomach.

In this study, we hypothesised that the presence of larger LNneg in the resection specimen is associated with improved survival in patients with locally advanced resectable OGAC irrespective of treatment modality. The aims of the present study were (i) to validate the prognostic relevance of LNneg size in patients with OGAC from two phase III trials, OE05^9^ [International Standard Randomised Controlled Trial Number (ISRCTN) 01852072] and ST03 (ISRCTN 46020948),^10^ with different preoperative and perioperative chemotherapy regimens and (ii) to investigate the relationship among LNneg size, primary tumour location and anthracotic pigment burden.

## Methods

### Patient cohorts

#### UK MRC OE05 trial

The UK MRC OE05 trial was a phase III trial in which patients with locally advanced resectable oesophageal or junctional adenocarcinoma were randomised to either receive two cycles of cisplatin/5-fluorouracil chemotherapy or receive four cycles of epirubicin, cisplatin and capecitabine (ECX) chemotherapy followed by surgery.^9^ Digital haematoxylin–eosin (H&E)–stained LNneg tissue sections were available from 652 resection specimens (87% of 751 patients who had a resection) for the current study [for Consolidated Standards of Reporting Trials (CONSORT) diagram, see Supplementary Figure S1A, available at https://doi.org/10.1016/j.esmoop.2026.108348].

#### UK MRC ST03 trial

The UK MRC ST03 trial was a phase II/III trial in which patients with gastric, junctional, or lower third oesophageal adenocarcinoma were randomised to receive either three cycles of ECX before and after surgery or three cycles of ECX + bevacizumab before and after surgery and further six doses of bevacizumab maintenance therapy.^10^ Digital H&E-stained slides containing LNnegs were available from 715 resection specimens (80% of 895 patients who had a resection) for the current study (for CONSORT diagram, see Supplementary Figure S1B, available at https://doi.org/10.1016/j.esmoop.2026.108348).

### LN segmentation and identification of the largest LNneg

**Figure 1:**
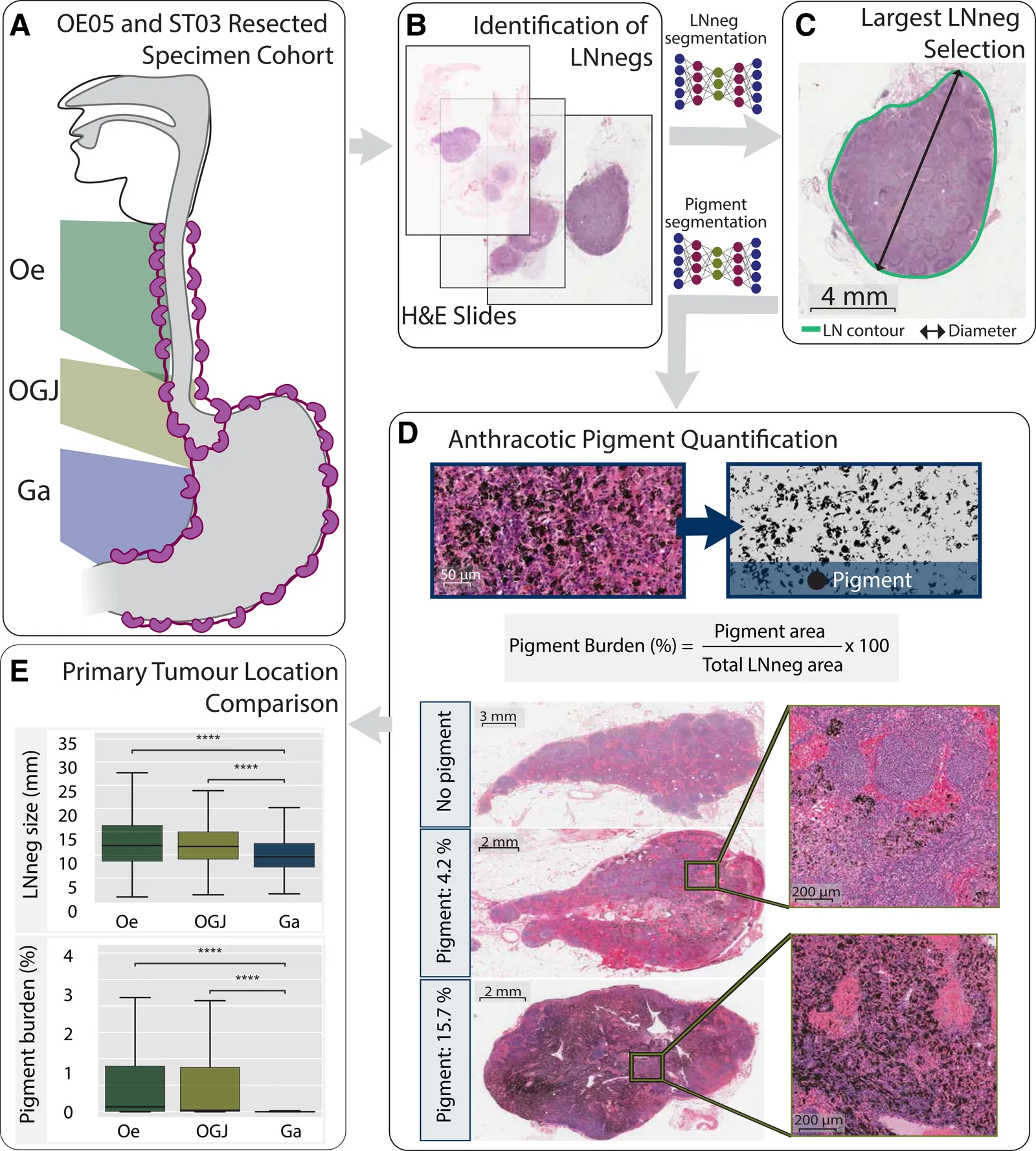
**Overview of the analytical workflow and results from primary tumour location–dependent comparisons of tumour-negative lymph node (LNneg) size and pigment burden.** (A) Resection specimens from patients enrolled in the OE05 and ST03 trials were analysed, including primary tumours from the Oe, OGJ, and Ga. (B) All available slides were analysed to identify LNnegs. (C) An LN segmentation model was applied to generate LN contours, and the longest LN axis was measured to identify the largest LNneg per patient. (D) Anthracotic pigment was quantified using a U-Net segmentation model, which we developed for this purpose. Representative H&E-stained LNnegs demonstrate heterogeneity in pigment burden, ranging from absence of pigment to involvement of up to 15.7% of the LNneg area. (E) Boxplots illustrating LNneg size and pigment burden differences between primary tumour location. Significant *P* values are annotated with asterisks: ∗*P* < 0.05, ∗∗*P* < 0.01, ∗∗∗*P* < 0.001, and ∗∗∗∗*P* < 0.0001.H&E, haematoxylin–eosin; LN, lymph node; LNneg, tumour-negative regional lymph node; Oe, oesophagus; OGJ, gastro-oesophageal junction; Ga, stomach.

A schematic overview of the study’s analytical workflow and the results from the comparison of LNneg size and pigment burden between primary tumour locations is shown in Figure 1. Digital slides with LNs were automatically identified and segmented using a deep learning–based model we developed previously.^11^ To ensure that only tumour-negative LNs and LNs without evidence of complete tumour regression were included in the current study, all segmented LNs were manually reviewed. At the same time, the automated segmentation was manually quality-controlled.

As in our previous study,^4^ we used the LNneg with the maximum long axis per patient for all analyses. This LN is referred to as LNneg from hereon. The LNneg long axis was computed using an image analysis software (scikit-image, S. van der Walt, South Africa) on the basis of ellipse fitting.^12^

### Anthracotic pigment segmentation

We developed a multiclass U-Net model^13^ for automatic segmentation of anthracotic pigment for the purpose of the current study (see Supplementary Text S1 for details, available at https://doi.org/10.1016/j.esmoop.2026.108348). To quantify the pigment within an LNneg (referred to as LNneg pigment burden), we calculated the percentage of LNneg area occupied by pigment.

### Statistical analyses

This research study was designed and reported in accordance with the Reporting Recommendations for Tumour Marker Prognostic Studies guidelines^14^ (see details in Supplementary Tables S1 and S2, available at available at https://doi.org/10.1016/j.esmoop.2026.108348). All analyses were carried out on the pooled cohort comprising patients from both trials (OE05 and ST03). Statistical analyses were carried out using SPSS Statistics software (version 30, IBM Corp, Armonk, NY) and Python libraries. Visualisation plots including boxplots and Kaplan–Meier plots were generated using Python 3.10 (Python Software Foundation, Wilmington, DE), seaborn, matplotlib and lifelines libraries. Forest plots were generated using R and Forest plot library (v 3.1.7; R Foundation for Statistical Computing, Vienna, Austria). Survival analysis, including Kaplan–Meier curves and Cox proportional hazards modelling, was carried out with Python’s lifelines library (v 0.30.0) (Python Software Foundation). Descriptive statistics including median, range of the largest LNneg size per patient and the clinicopathological variable distribution across both clinical trials were calculated. For the comparisons presented in boxplots, statistical significance was assessed using Kruskal–Wallis test and the Mann–Whitney *U* tests as appropriate. The following clinicopathological characteristics were available from the MRC clinical trial unit database or from the central pathology review and considered for analyses: sex, primary tumour location at diagnosis as determined by endoscopy [oesophagus (Oe), OGJ, or stomach (Ga)], pathological depth of invasion (ypT, TNM classification seventh ed), pathological LN status [ypN, to increase statistical power, we compared ypN0 (no LN metastasis) versus ypN1+ (any number of LN metastasis)], Mandard tumour regression grade (TRG), pathological resection margin status (R0 versus R1), type of recurrence after surgery (local only, distant only, both local and distant, or no recurrence), lymphovascular invasion status (present versus absent), histological subtype (diffuse versus intestinal versus mucinous), and LNneg pigment burden.

For regression analyses, ypT was dichotomised as ypT0-2 vs ypT3-4, and Mandard TRG as TRG1-3 vs TRG4-5. The lymph node ratio (LNratio; number of metastatic LNs divided by the total number of resected LNs) was calculated for patients with stage ypN1+ cancer only. The LNratio was dichotomised into low and high LNratio using the median value as cut-off.

Univariable ordinary least squares (OLS) regression models were fitted for every clinicopathological variable available, using continuous LNneg size (millimetres) as the dependent variable. To account for potential differences in LNneg size between patients from OE05 and ST03 clinical trials, the trial was included as a covariate in all regression models. For categorical variables, regression coefficients represent the absolute difference in mean LNneg size between the comparison group and the reference category. For continuous variables (pigment burden), coefficients represent the expected change in LNneg size per one-unit increase in the variable. Univariable effect estimates are reported together with 95% confidence intervals (CIs), which in the OLS framework correspond to parametric Wald-type intervals for the expected mean effect.

To evaluate the associations of clinicopathological variables with LNneg size while adjusting for potential confounders, a multivariable OLS model was subsequently fitted. Variables demonstrating evidence of association in univariable analyses and/or considered clinically relevant a priori were included in the multivariable model. Model fitting was carried out using the statsmodels OLS framework (version 0.14.5) in Python (Python Software Foundation).^15^ Results are presented as Forest plots showing adjusted effect estimates with corresponding 95% CI and *P* values.

OS and progression-free survival (PFS) from surgery were analysed using Kaplan–Meier curves and log-rank statistics. Hazard ratios (HRs) and corresponding 95% CIs were estimated using univariable Cox proportional hazards models. All survival analyses were carried out dichotomising LNneg size at a 10 mm threshold (≤10 mm versus >10 mm). A 10 mm threshold was used because this is also used by radiologists to classify an LN as enlarged.^16^ To assess whether the association between OS and LNneg size was linear, LNneg size was additionally modelled as a continuous variable using restricted cubic spline terms (4 degrees of freedom) within Cox proportional hazards regression models, with estimated 5-year OS probabilities being derived from the fitted model. Both univariable and multivariable spline models demonstrated a nonlinear association with survival, improving as LNneg size increased up to 10-12 mm, after which the effect plateaued. These findings support the appropriateness of the dichotomised approach used in the primary analyses (Supplementary Figure S2, available at https://doi.org/10.1016/j.esmoop.2026.108348).

Cox proportional hazards models were stratified by trial (lifelines ‘strata’ option), allowing for different baseline hazard functions between trials while assuming that the relative effect of LNneg size on survival did not differ by trial. Univariable Cox regression analyses were first carried out to assess the association of ypT, ypN, age, sex, Mandard TRG, primary tumour location, lymphovascular invasion status, histological subtype, and treatment arm with OS or PFS. Variables showing statistical significance in univariable analyses were subsequently included as covariates in a multivariable Cox regression model assessing the prognostic effect of LNneg size dichotomised at 10 mm. *P* values <0.05 were considered significant.

## Results

### Patient cohort characteristics

This study included a total of 1367 patients with locally advanced resectable OGAC: 652 from the OE05 trial and 715 from the ST03 trial (for clinicopathological characteristics, see Table 1). In both cohorts, the majority of patients were male, and the age distribution was similar [OE05: median 62 years (range 30-80 years), ST03: median 63 years (range 31-79 years)]. The OE05 trial included predominantly patients with lower oesophageal adenocarcinoma (*n* = 452, 77.5%), whereas ST03 included predominantly patients with gastric adenocarcinoma (*n* = 380, 53%). The maximum long-axis diameter of the tumour-negative LN (LNneg size) was slightly different between trials [OE05 versus ST03: median 11.9 mm (range 0.9-34.7 mm) versus 10.4 mm (range 1.4-33.2 mm), respectively]. The LNneg pigment burden differed significantly between trials [OE05 versus ST03: median 0.07% (range 0%-18.5%) versus 0.002% (range 0%-13.4%), respectively, *P* < 0.0001]. All statistical analyses presented below were carried out by pooling the data from both trials, adjusting for trial as a covariate in OLS models and as a stratification factor in Cox models.

**Table 1:** Clinicopathological characteristics of the patient cohorts stratified by trial

Characteristics	Total		OE05		ST03	
	*n*	%	*n*	%	*n*	%
Sex						
Male	1157	84.6	582	89.3	575	80.4
Female	210	15.4	70	10.7	140	19.6
Age						
<70 years	1137	83.2	553	84.8	584	81.7
≥70 years	230	16.8	99	15.2	131	18.3
Primary tumour location						
Oesophageal	628	48.4	452	77.5	176	24.6
Oesophagogastric junction	290	22.3	131	22.5	159	22.2
Gastric	380	29.3	0	0	380	53.1
Depth of invasion (ypT)						
0	66	4.9	27	4.1	39	5.5
1	140	10.3	59	9.1	81	11.5
2	198	14.6	83	12.7	115	16.3
3	775	57.1	409	62.8	366	51.8
4	179	13.2	73	11.2	106	15
Type of disease recurrence						
Local	148	13.5	71	13.7	77	13.4
Distant	225	20.5	118	22.7	107	18.6
Local + distant	183	16.7	124	23.8	59	10.3
No recurrence	539	49.2	207	39.8	332	57.7
Total LN yield						
<15 LNs	206	15.3	94	14.5	112	16
15-24 LNs	441	32.7	203	31.3	238	34
≥25 LNs	703	52.1	352	54.2	351	50.1
Histological subtype						
Diffuse	195	14.3	33	5.1	162	22.8
Intestinal	1041	76.5	577	88.8	464	65.4
Mucinous	124	9.1	40	6.2	84	11.8
Lymphovascular invasion						
No	725	53.4	357	55	368	52
Yes	632	46.6	292	45	340	48
Resection margin status						
R0 (negative)	882	65.5	357	55.6	525	74.6
R1 (positive)	464	34.5	285	44.4	179	25.4
Mandard tumour regression grade						
1	66	4.9	27	4.2	39	5.5
2	71	5.2	27	4.2	44	6.2
3	250	18.5	81	12.5	169	23.9
4	646	47.7	270	41.7	376	53.3
5	320	23.7	242	37.4	78	11
LN status (ypN)						
0 (no metastasis and no evidence of tumour regression)	397	29.2	176	27	221	31.3
0 (no metastasis but some LN with evidence of tumour regression)	110	8.1	41	6.3	69	9.8
1+	851	62.7	434	66.7	417	59
LN size (mm)						
≤10mm	560	41	233	35.7	327	45.7
>10mm	807	59	419	64.3	388	54.3

Numbers may not always add up to 100% (*n* = 1367) owing to missing values or rounding.

LN, lymph node; LNneg, tumour-negative regional LN.

### Association between LNneg size and clinicopathological characteristics

LNneg size was significantly related to primary tumour location (*P* < 0.0001; Oe: *n* = 628, median 12.0 mm (range 0.9-33.7 mm); OGJ: *n* = 290, median 11.8 mm (range 1.4-34.7 mm); Ga, *n* = 380, median 9.6 mm (range 1.6-31.0 mm)]. Pairwise comparisons showed that LNnegs from patients with oesophageal or OGJ cancers were larger than those from patients with gastric cancers (Oe versus Ga and OGJ versus Ga, all *P* < 0.0001, Figure 1E). Furthermore, LNneg pigment burden differed by primary tumour location, with higher pigment burden observed in Oe and OGJ compared with Ga (Oe versus Ga and OGJ versus Ga, all *P* < 0.0001; Figure 1E).

**Figure 2:**
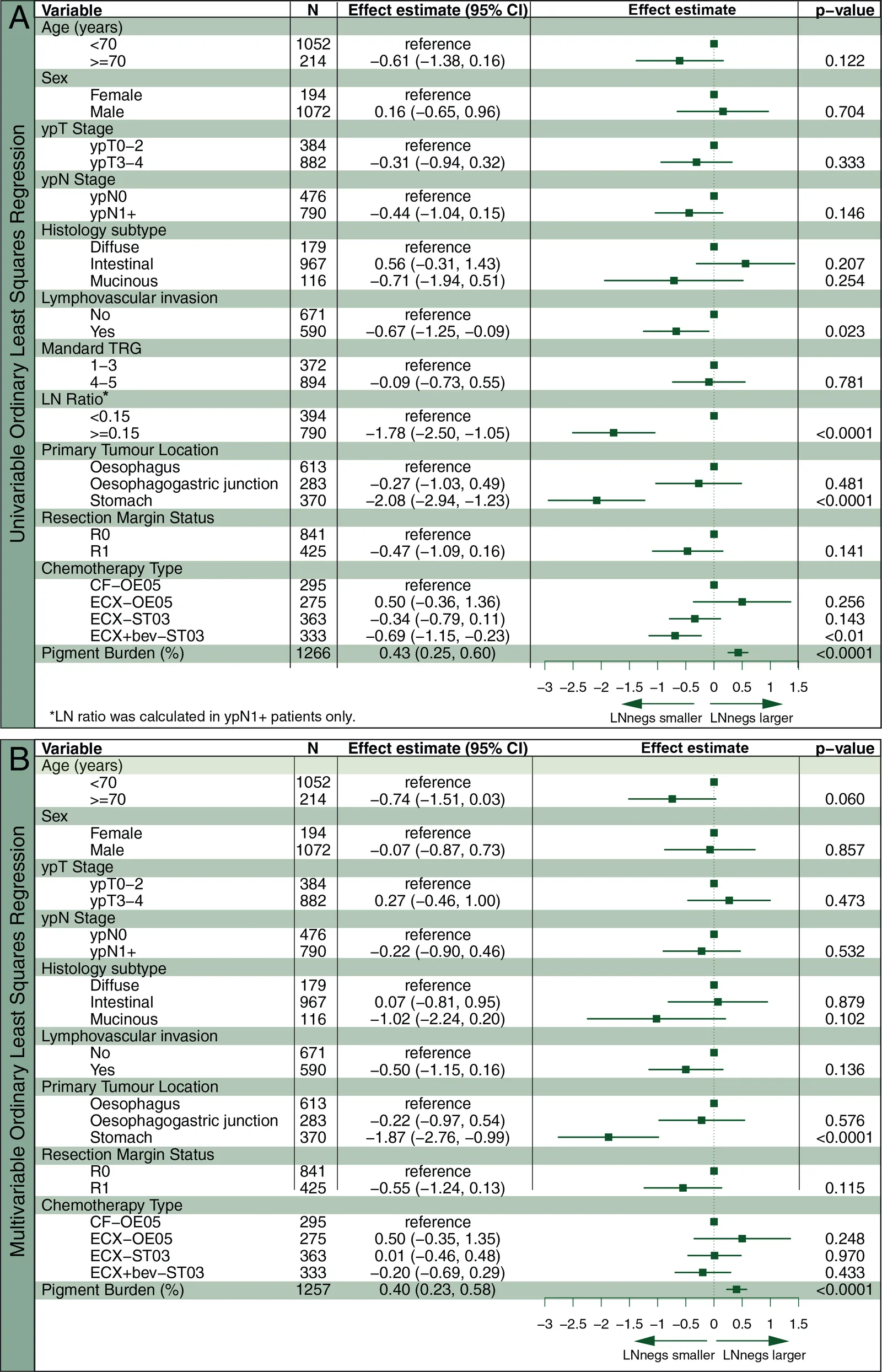
**Forest plots of linear regression analyses evaluating associations between clinicopathological variables and tumour-negative lymph node (LNneg) size.** Effect estimates and 95% CIs represent ordinary least squares (OLS) β coefficients, indicating the mean difference in LNneg size relative to the reference category. (A) Univariable analysis. (B) Multivariable analysis.CF, cisplatin and 5-fluorouracil; CI, confidence interval; ECX, epirubicin, cisplatin and capecitabine; ECX+bev, ECX plus bevacizumab; LN, lymph node; TRG, tumour regression grade.

We used OLS regression analysis to investigate the association between clinicopathological factors and LNneg size. Univariable OLS regression analysis results are illustrated in Figure 2A. Patients with gastric cancers had smaller LNnegs compared with patients with oesophageal cancers (*P* < 0.0001). LNneg pigment burden was associated with larger LNnegs (*P* < 0.0001). In patients with stage ypN1+ cancer, a high LNratio (≥0.15) was related to smaller LNneg compared with those with a low LNratio (*P* < .0001). After multivariable adjustment, primary tumour location and LNneg pigment burden remained independently associated with LNneg size (Figure 2B). Patients with lymphovascular invasion had smaller LNnegs compared with patients without lymphovascular invasion (*P* = 0.023). However, this association was no longer significant after adjustment for clinicopathological confounders in multivariable OLS (Figure 2A and B).

### Association of LNneg size with OS and PFS

**Figure 3:**
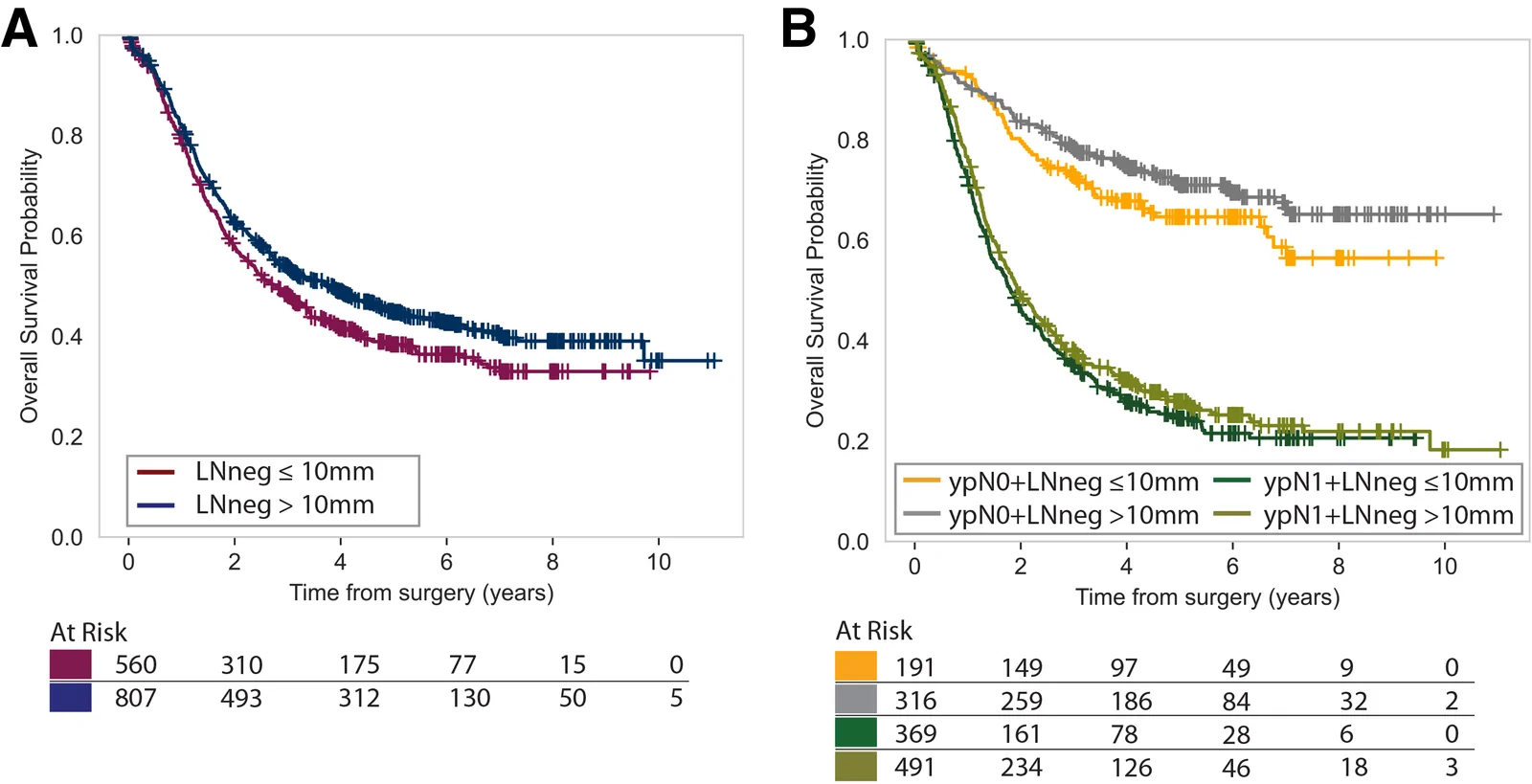
**Kaplan–Meier curves of overall survival stratified by tumour-negative lymph node (LNneg) size.** (A) Overall survival (OS) in the pooled trial cohort stratified by LNneg size (≤10 mm versus >10 mm). Patients with LNneg >10 mm demonstrated improved OS compared with those with LNneg ≤10 mm [hazard ratio (HR) 0.81, 95% confidence interval (CI) 0.70-0.93, log-rank *P* = 0.0147]. (B) No statistically significant difference in OS according to LNneg size was observed after grouping patients by lymph node status [large vs small LNnegs in ypN0 (HR 0.76, 95% CI 0.55-1.04, log-rank *P* = 0.107) and ypN1+ (HR 0.87, 95% CI 0.74-1.03, log-rank *P* = 0.239)]. HRs and 95% CIs were derived from univariate Cox proportional hazards models stratified by trial.

LNneg size was dichotomised as small (≤10 mm) or large (>10 mm) using a clinically established radiological LN size cut-off,^16^ which was at the same time close to the whole cohort’s LNneg size median (11 mm). In Kaplan–Meier analyses, patients with large LNnegs had a better OS (HR 0.81, 95% CI 0.70-0.93, log-rank *P* = 0.0147; Figure 3A) and better PFS (HR 0.81, 95% CI 0.70-0.92, log-rank *P* = 0.0092; Supplementary Figure S3A, available at https://doi.org/10.1016/j.esmoop.2026.108348). In univariable Cox OS analysis, larger LNneg size was associated with better OS (*P* < 0.01, Table 2). In multivariable Cox OS analysis, LNneg size remained significant when primary tumour location, Mandard TRG, ypN, ypT, histological subtype, lymphovascular invasion, and sex were included in the model (HR 0.82, 95% CI 0.7-0.95, *P* < 0.01; Table 2). Similar associations were observed for PFS, Kaplan–Meier plots (Supplementary Figure S3, available at https://doi.org/10.1016/j.esmoop.2026.108348) and Cox regression analysis results (Supplementary Table S3, available at https://doi.org/10.1016/j.esmoop.2026.108348).

**Table 2:** Univariable and multivariable Cox regression analysis of overall survival

Variable	Univariable			Multivariable		
	HR	95% CI	*P* value	HR	95% CI	*P* value
Age (≥70 vs <70 years)	0.99	0.82-1.19	0.897	-	-	-
Sex (male vs female)	1.39	1.12-1.73	<0.01	1.42	1.12-1.79	<0.01
Pigment burden (% LNneg area)	0.99	0.95-1.03	0.713	-	-	-
Primary tumour location						
Gastric vs Oe	0.77	0.62-0.95	0.015	0.88	0.69-1.13	0.314
Oesophagogastric junction vs Oe	1.3	1.1-1.54	<0.01	1.13	0.94-1.37	0.203
Mandard TRG (4-5 vs 1-3)	2.13	1.77-2.55	<0.0001	1.24	1.01-1.51	0.037
Chemotherapy regimen						
ECX-OE05 vs CF-OE05	0.9	0.74-1.09	0.284	-	-	-
ECX-ST03 vs CF-OE05	0.96	0.78-1.18	0.696	-	-	-
ECX+bev-ST03 vs CF-OE05	1.04	0.85-1.28	0.696	-	-	-
Histological subtype						
Intestinal vs diffuse	0.78	0.66-0.93	<0.01	0.85	0.71-1.02	0.083
Mucinous vs diffuse	0.91	0.71-1.18	0.479	-	-	-
LNneg size (>10 mm vs ≤10 mm)	0.81	0.7-0.93	<0.01	0.82	0.7-0.95	<0.01
ypT (ypT 3-4 vs ypT0-2)	3.27	2.69-3.98	<0.0001	1.84	1.48-2.29	<0.0001
Lymphovascular invasion (yes vs no)	2.44	2.11-2.82	<0.0001	1.52	1.29-1.79	<0.0001
ypN (ypN1+ vs ypN0)	3.49	2.93-4.17	<0.0001	2.3	1.89-2.79	<0.0001

bev, bevacizumab; CF, cisplatin, 5-fluorouracil; CI, confidence interval; ECX, epirubicin, cisplatin, capecitabine; HR, hazards ratio; LNneg, tumour-negative regional lymph node; Oe, oesophagus; TRG, tumour regression grade.

The predicted 5-year OS probability for patients with larger LNnegs (>10 mm) was 43% (95% CI 41%-48%), compared with 38% (95% CI 34%-43%) for patients with smaller LNnegs, corresponding to an absolute predicted OS difference of 5 percentage points at the 5-year cut-off.

As in our previous study in the OE02 trial patients, we next evaluated the relationship between LNneg size and survival, stratifying patients by ypN status.^4^ As LNneg size was similar in ypN0 with and without evidence of tumour regression (see distribution boxplot in Supplementary Figure S4A, available at https://doi.org/10.1016/j.esmoop.2026.108348), all patients with ypN0 status were analysed together. There was no significant difference in OS comparing large and small LNneg size, neither in patients with stage ypN0 cancer nor in those with stage ypN1+ cancer (Kaplan–Meier plot provided in Figure 3B).

## Discussion

Clinical and pathological LN status is the strongest prognostic factor in patients with locally advanced resectable oesophagogastric cancer.^2^ The number of tumour-positive regional LNs is part of the TNM classification owing to its well-established association with survival. In contrast, LNnegs have received far less attention as a potential clinically useful prognostic biomarker.

In this large, pooled analysis of two phase III trials (OE05 and ST03), we validated LNneg size as an independent prognostic biomarker in 1367 patients with locally advanced OGAC treated with preoperative or perioperative cytotoxic chemotherapy. Patients with larger LNnegs had improved OS and PFS independent of established clinicopathological prognostic factors. A notable finding of the current study was the strong inverse relationship between LNneg size and LNratio in patients with stage ypN1+ cancer, whereby a greater metastatic tumour burden (increased LNratio) was associated with smaller LNnegs, suggesting that LNneg size may reflect systemic or regional LN immune competence. Moreover, larger LNnegs were associated with lower recurrence rates, providing further support for the hypothesis that LNneg enlargement may represent a surrogate marker of a more effective host antitumour response rather than representing anatomical variation alone.

This study is the first to demonstrate that histological LNneg size and anthracotic pigment burden vary by primary tumour location, likely reflecting anatomical and biological variation in the lymphatic drainage. LNnegs from oesophageal or junctional tumours were larger and contained more pigment than those from gastric tumours. These histological differences in LNneg size mirror radiological observations in healthy populations, in whom mediastinal LNs are larger than upper abdominal LNs.^17^^,^^18^

Our findings are consistent with prior evidence from the OE02 trial oesophageal cancer cohort^4^ and extend these observations to patients with junctional or gastric adenocarcinoma treated with diverse preoperative or perioperative chemotherapy regimens. This indicates that LNneg size is a robust and reproducible novel prognostic biomarker across different tumour locations within the upper gastrointestinal tract and across different treatment modalities. LNneg size is a simple histomorphological parameter that can easily be integrated into standard pathology workflows or measured automatically using digital slide analysis pipelines.^19^, ^20^, ^21^ Its independent prognostic value appears independent of ypN stage and may therefore help refine risk stratification of patients with ypN0 or ypN1 oesophagogastric cancer, particularly in the postneoadjuvant setting, in which conventional pathologic indicators (ypT, ypN, or TRG) may be less discriminative.^22^ The lack of statistical significance within ypN strata in univariate survival likely reflects reduced power rather than the absence of a biological effect.

When considering 5-year OS, we observed an absolute improvement of ∼5 percentage points. Although modest in magnitude, such a difference may still represent a clinically meaningful benefit in the curative setting, in which OS represents a definitive endpoint reflecting true life extension. Comparable effect sizes have previously been sufficient to influence clinical practice in oncology, including the OE02 trial (∼6% OS gain),^5^ the MOSAIC trial (∼5%),^23^^,^^24^ and the BILCAP trial (∼5%-6%).^25^ In upper gastrointestinal cancer, the FLOT4 trial demonstrated an ∼9% improvement in 5-year OS,^26^ further supporting the concept that even single-digit absolute survival gains can be clinically meaningful and practice-changing.

Whether LNneg size after cytotoxic chemotherapy can also predict which patients will benefit from adjuvant therapy and/or immunotherapy in particular remains to be investigated.

This study has some limitations. This study is retrospective in nature using material from patients recruited to two phase III clinical trials. Although surgical procedures and resection specimen handling were prespecified in the trial protocol, only a few trial centres recorded the anatomical LN station number for the dissected LNs. We therefore used the pretreatment primary tumour location as a proxy for LNneg location, acknowledging the potential for misclassification, and were unable to draw LN station–specific conclusions. This limitation is particularly relevant for OGJ cancers, which may be treated by either oesophagectomy or extended gastrectomy, each involving distinct lymphadenectomy fields. Accordingly, our analyses focused primarily on differences between LNnegs derived from oesophageal and those derived from gastric primary tumours. Another limitation of this study is the possibility that a single LN may have been represented multiple times if sectioned into several tissue fragments, potentially affecting the accuracy of the true LN size assessment. To minimise this limitation, the analysis was restricted to the single largest nonmetastatic LN fragment per patient, which was used as a representative LNneg. This reduced potential selection bias related to the choice of a particular LN section.

We were unable to investigate the LN microarchitecture in the current study and thus cannot comment at this moment whether LN enlargement reflects specific changes in the LN microarchitecture, such as germinal centre expansion or lymphocyte density. As LNneg size was measured in LNs from resection specimens, this limits its use for treatment decisions at the time of diagnosis or in patients with unresectable disease.

Furthermore, environmental factors such as tobacco smoking, occupational dust exposure, and air pollution may contribute to LN pigment burden; however, these data were not available in the present study. This limits any inference regarding the potential clinical or prognostic significance of pigment deposition. Therefore, we report pigment burden differences by primary tumour location as an exploratory finding, reflecting anatomical differences in lymphatic drainage pathways, without speculating on the origin or biological implications of these pigment particles.

Further studies are warranted to explore whether modern imaging (computed tomography, magnetic resonance imaging and ultrasound) combined with deep learning–based image analysis approaches is able to identify tumour-negative LNs with high confidence and provide long-axis measurements.

In summary, this study established LNnegs size as a robust and reproducible novel prognostic biomarker in patients with locally advanced OGAC treated with preoperative or perioperative chemotherapy. By shifting the focus from metastatic LNs to the biological significance of tumour-negative LNs, our findings support the concept that regional LN morphology may be associated with the host antitumour interaction and immune competence. Prospective validation in contemporary treatment cohorts, including patients receiving modern perioperative regimens such as FLOT combined with immune checkpoint inhibitors,^27^ as well as chemoradiotherapy and/or immunotherapy-based treatment strategies, is warranted to determine whether the prognostic relevance of LNneg number and LNneg size remains consistent in the era of perioperative immunotherapy. Future studies should integrate quantitative assessment of LNneg size with detailed microarchitectural analysis and immune cell phenotyping to better elucidate the immune biological mechanisms underlying the prognostic effect of LNneg size. If confirmed prospectively, LNneg size could complement TNM staging and contribute to more individualised perioperative risk stratification and treatment decision making in patients with upper gastrointestinal cancer.
