# Supplementary material for: Validation of tumour-negative lymph node size as a novel independent prognostic biomarker in oesophagogastric adenocarcinoma: post hoc analysis of the UK MRC OE05 and ST03 randomised trials
Source: ESMO Open. 2026 Aug 12;11(9):108348. doi: 10.1016/j.esmoop.2026.108348 (PMC13488028; doi:10.1016/j.esmoop.2026.108348)
Supplement: Supplementary file 1 [file mmc1.docx]

Supplementary Materials

**Title**: Validation of tumour-negative lymph node size as novel independent prognostic biomarker in oesophagogastric adenocarcinoma: post-hoc analysis of the UK MRC OE05 and ST03 randomised trials

Elzbieta Budginaite^1,2^, Maximilian Kloft^1,3,4^, Derek R. Magee^5^, Shahab Jolani^6^, Thorsten O. Goetze^3,4^, Matthew G Nankivell^7^, Ruth E. Langley^7^, David Cunningham^8^, William H. Allum^9^, Henry C. Woodruff^2^, Heike I. Grabsch^1,10^*

**Table of contents**

| Supplementary table S1 | REMARK checklist |
| --- | --- |
| Supplementary table S2 | REMARK profile |
| Supplementary figure S1 | Patient flow diagrams for OE05 and ST03 clinical trials |
| Supplementary figure S2 | OS by LNneg modelled as a continuous variable restricted cubic splines |
| Supplementary text S1 | Method for pigment segmentation |
| Supplementary figure S3 | PFS Kaplan Meier figure |
| Supplementary table S3 | PFS Cox survival analysis |
| Supplementary figure S4 | Boxplots for per-nodal stage distribution for LNneg size and nodal pigment burden. |

**Supplementary table S1**: REMARK checklist.

| **Item to be reported** | | **Page no.** |
| --- | --- | --- |
| **INTRODUCTION** | |  |
| 1 | State the marker examined, the study objectives, and any pre-specified hypotheses. | 3-4 |
| **MATERIALS AND METHODS** | |  |
| *Patients* | |  |
| 2 | Describe the characteristics (e.g., disease stage or co-morbidities) of the study patients, including their source and inclusion and exclusion criteria. | 5 |
| 3 | Describe treatments received and how chosen (e.g., randomised or rule-based). |  |
| *Specimen characteristics* | |  |
| 4 | Describe type of biological material used (including control samples) and methods of preservation and storage. | 5 |
| *Assay methods* | |  |
| 5 | Specify the assay method used and provide (or reference) a detailed protocol, including specific reagents or kits used, quality control procedures, reproducibility assessments, quantitation methods, and scoring and reporting protocols. Specify whether and how assays were performed blinded to the study endpoint. | 5-6 |
| *Study design* | |  |
| 6 | State the method of case selection, including whether prospective or retrospective and whether stratification or matching (e.g., by stage of disease or age) was used. Specify the time period from which cases were taken, the end of the follow-up period, and the median follow-up time. | 5-8 |
| 7 | Precisely define all clinical endpoints examined. |  |
| 8 | List all candidate variables initially examined or considered for inclusion in models. |  |
| 9 | Give rationale for sample size; if the study was designed to detect a specified effect size, give the target power and effect size. *(all available samples were analyzed)* |  |
| *Statistical analysis methods* | |  |
| 10 | Specify all statistical methods, including details of any variable selection procedures and other model-building issues, how model assumptions were verified, and how missing data were handled. | 6-7 |
| 11 | Clarify how marker values were handled in the analyses; if relevant, describe methods used for cutpoint determination. |  |
| **RESULTS** | |  |
| *Data* | |  |
| 12 | Describe the flow of patients through the study, including the number of patients included in each stage of the analysis (a diagram may be helpful) and reasons for dropout. Specifically, both overall and for each subgroup extensively examined report the numbers of patients and the number of events. | 8 |
| 13 | Report distributions of basic demographic characteristics (at least age and sex), standard (disease-specific) prognostic variables, and tumour marker, including numbers of missing values. | 8-9 |
| *Analysis and presentation* | |  |
| 14 | Show the relation of the marker to standard prognostic variables. | 9-10 |
| 15 | Present univariable analyses showing the relation between the marker and outcome, with the estimated effect (e.g., hazard ratio and survival probability). Preferably provide similar analyses for all other variables being analyzed. For the effect of a tumour marker on a time-to-event outcome, a Kaplan-Meier plot is recommended. | 10 |
| 16 | For key multivariable analyses, report estimated effects (e.g., hazard ratio) with confidence intervals for the marker and, at least for the final model, all other variables in the model. | 10 |
| 17 | Among reported results, provide estimated effects with confidence intervals from an analysis in which the marker and standard prognostic variables are included, regardless of their statistical significance. | 10 |
| 18 | If done, report results of further investigations, such as checking assumptions, sensitivity analyses, and internal validation. | 10 |
| **DISCUSSION** | |  |
| 19 | Interpret the results in the context of the pre-specified hypotheses and other relevant studies; include a discussion of limitations of the study. | 10-13 |
| 20 | Discuss implications for future research and clinical value. | 12-13 |

**Supplementary table S2**: REMARK profile.

| Study marker | Remarks | | | |
| --- | --- | --- | --- | --- |
| Marker | LNneg size (≤10mm vs >10mm)  Pigment burden (%) – % area of LNneg covered in pigment. | | | |
| Further variables | Sex, ypT stage, ypN stage, location of primary tumour, resection margin status, Mandard tumour regression grade, type of recurrence, lymphovascular invasion, histological subtype. | | | |
| Patients | N | Remarks | | |
| Assessed for eligibility | 1960 |  | | |
| Excluded | 593 | Surgery not performed (n=259), no slides from the resection specimen received (n=220), no slides with LNnegs received (n=114) | | |
| Included | 1367 | Oe05 trial: 652; ST03 trial: 715 | | |
| With outcome events | 774 (OS)  827 (PFS) | Overall survival: death from any cause  Progression-free survival: evidence of disease progression or death from any cause | | |
| Statistical analyses of survival outcomes | | | | |
| Analysis | Patients | Events | Variables considered | Results |
| Overall survival | | | | |
| A1: Univariable | 1367 | 774 | Age at diagnosis, sex, chemotherapy regimen, primary tumour location, ypT, ypN, Mandard tumour regression grade, lymphovascular invasion, histological subtype. pigment burden, LNneg size (>10mm) | Figure 3  Table 2 |
| A2: Multivariable | 1283 | 720 | sex, primary tumour location, ypT, ypN, Mandard tumour regression grade, lymphovascular invasion, histological subtype. pigment burden, LNneg size (>10mm) | Table 2 |
| Progression-free survival | | | | |
| B1: Univariable | 1367 | 827 | Age at diagnosis, sex, chemotherapy regimen, primary tumour location, ypT, ypN, Mandard tumour regression grade, lymphovascular invasion, histological subtype. pigment burden, LNneg size (>10mm) | Supplementary figure S2 and Supplementary table 1 |
| B2: Multivariable | 1283 | 768 | Age at diagnosis, sex, chemotherapy regimen, primary tumour location, ypT, ypN, Mandard tumour regression grade, lymphovascular invasion, histological subtype. pigment burden, LNneg size (>10mm) | Supplementary table 1 |

**Supplementary figure S1**: CONSORT diagrams for OE05 (A) and ST03 (B) clinical trials illustrating number of patients randomized for each clinical trial (top) and number of patients included in the current study (bottom).

**Supplementary Figure S2. Predicted 5-year overall survival probabilities according to LNneg size modelled as a continuous variable using restricted cubic splines in Cox regression models.**

Predicted 5-year overall survival (OS) probabilities with corresponding confidence intervals derived from univariable (A) and multivariable (B) Cox proportional hazards models using LNneg size as a continuous spline term (4 degrees of freedom). The multivariable model was adjusted for the same clinicopathological confounders as reported in Table 2 in the manuscript. Both univariable and multivariable models demonstrated a non-linear association between LNneg size and predicted OS probability, showing a monotonous increase to approximately 10-12 mm, and plateauing for larger LNneg sizes.

**Methods S1.** Pigment segmentation

The pigment segmentation model was designed to discriminate three classes: black pigment, H&E-stained tissue, and background (tissue-absent slide areas). We selected a lightweight U-Net architecture with a minimal number of hidden layers to limit excessive downsampling, as the small size of pigment speckles may not be preserved if spatial resolution is reduced too aggressively in the encoder–decoder pathway.

For model development, 40 pigment-containing slides from the OE02 trial were selected. A rule-based colour thresholding approach was used to generate rough pigment area annotations. H&E tissue and background masks were obtained using tissue segmentation results from the GrandQC model [1]. The training set was expanded using the same augmentation strategies described above.

The model was trained for 150 epochs on a mixture of 512×1024 pixel patches extracted at either 40× or 20× magnification, yielding a total of 4,900 training tiles. During inference, pigment segmentation was performed at the highest available magnification (40× or 20×, depending on the slide).

To quantify pigment burden (%), the percentage of predicted LNneg area occupied by pigment was calculated.

1. Weng, Z., et al., GrandQC: A comprehensive solution to quality control problem in digital pathology. Nature Communications, 2024. 15(1): p. 10685.

**Supplementary figure S3:** **Kaplan–Meier curves of progression-fee survival according to tumour-negative lymph node (LNneg) size.**

(**A**) Progression-free survival (PFS) in the pooled trial cohort stratified by LNneg size (≤10 mm vs >10 mm). Patients with LNneg >10mm demonstrated improved PFS compared to those with LNneg ≤10 mm (HR 0.80, 95% CI 0.70-0.92; log-rank p=0.0092).

(**B**) PFS stratified by LNneg size and lymph node status (ypN0 vs ypN1+). ypN0 patients with large LNneg (>10mm) showed improved PFS compared to ypN0 patients with small LNneg (≤10 mm) (HR 0.73, 95% CI 0.54–0.98; log-rank p=0.0468). No difference in PFS was observed between ypN1+ patients with large LNnegs compared to those with small LNnegs (HR 0.89, 95% CI: 0.76-1.04, log-rank p=0.326). Hazard ratios (HRs) and 95% confidence intervals (CIs) were derived from univariable Cox proportional hazards models stratified by trial.

**Supplementary table S3**. Univariable and multivariable Cox progression-free survival analysis.

|  | **Univariable analysis** | | |  | **Multivariable analysis** | | |
| --- | --- | --- | --- | --- | --- | --- | --- |
|  | **HR** | **95% CI** | **p-value** |  | **HR** | **95% CI** | **p-value** |
| **Age** (≥70 vs <70 yrs) | 0.96 | 0.8-1.16 | 0.694 |  |  | - |  |
| **Pigment Burden (%)** | 0.99 | 0.95-1.03 | 0.63 |  |  | - |  |
| **Primary tumour location**:  Gastric vs Oesophageal | 0.79 | 0.64-0.96 | **0.02** |  | 0.88 | 0.7-1.11 | 0.281 |
| Oesophagogstric juction vs Oesophageal | 1.29 | 1.1-1.52 | **p<0.01** |  | 1.13 | 0.94-1.35 | 0.207 |
| **Mandard TRG** (4-5 vs 1-3) | 2.13 | 1.79-2.53 | **p<0.0001** |  | 1.27 | 1.05-1.54 | **0.015** |
| **Chemotherapy regimen**  ECX-OE05 vs CF-OE05 | 0.87 | 0.73-1.05 | 0.16 |  |  | - |  |
| ECX-ST03 vs CF-OE05 | 0.97 | 0.79-1.18 | 0.757 |  |  | - |  |
| ECX+bev-ST03 vs CF-OE05 | 1.03 | 0.84-1.26 | 0.757 |  |  | - |  |
| **Histological subtype:**  Intestinal vs Diffuse | 0.8 | 0.68-0.94 | **p<0.01** |  | 0.89 | 0.75-1.06 | 0.196 |
| Mucinous vs Diffuse | 0.92 | 0.72-1.17 | 0.503 |  |  | - |  |
| **LNneg size** (>10mm vs ≤10mm) | 0.8 | 0.7-0.92 | **p<0.01** |  | 0.81 | 0.7-0.94 | **p<0.01** |
| **ypT** (ypT 3-4 vs ypT0-2) | 3.21 | 2.67-3.87 | **p<0.0001** |  | 1.88 | 1.53-2.31 | **p<0.0001** |
| **Sex** (Male vs Female) | 1.26 | 1.03-1.55 | **0.026** |  | 1.26 | 1.02-1.57 | **0.036** |
| **Lymphovascular invasion** (Yes vs No) | 2.38 | 2.07-2.73 | **p<0.0001** |  | 1.5 | 1.28-1.76 | **p<0.0001** |
| **ypN** (ypN1+ vs ypN0) | 3.3 | 2.79-3.9 | **p<0.0001** |  | 2.18 | 1.81-2.63 | **p<0.0001** |

**Supplementary figure S4**: Boxplots illustrating largest LNneg size (mm) (**A**) and anthracotic pigment burden (**B**) distributions stratified by ypN stage. No statistically significant differences were observed between groups. Abbreviations: ypN0+regr: ypN0 patients with LNneg containing the evidence of tumour regression; ypN0-regr: ypN0 patients with no sign of tumour regression in LNneg.
